# Noise Cancellation Effects in Integrated Photonics with Wilkinson Power Dividers

**DOI:** 10.1021/acsphotonics.2c01675

**Published:** 2023-04-12

**Authors:** Angel Ortega-Gomez, Osmery Hernández, Douglas Oña, Carlos Biurrun-Quel, Carlos del Río, Iñigo Liberal

**Affiliations:** Department of Electrical, Electronic and Communications Engineering, Institute of Smart Cities (ISC), Pamplona 31006, Spain

**Keywords:** Wilkinson power divider, noise performance, thermal emission, integrated optics, ring resonator

## Abstract

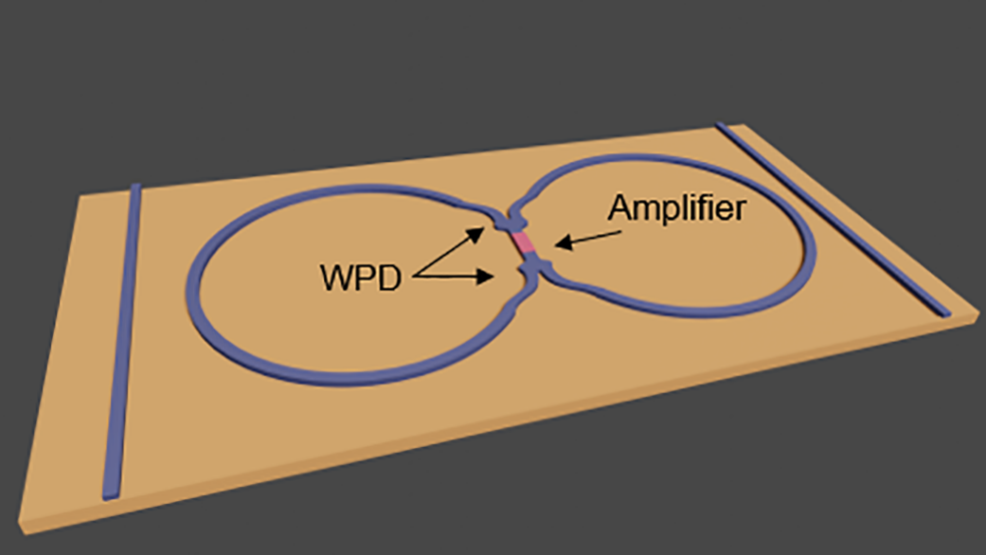

Wilkinson power dividers (WPDs) are a popular element in RF and microwave technologies known for providing isolation capabilities. However, the benefits that WPDs could offer to integrated photonic systems are far less studied. Here, we investigate the thermal emission from and the noise performance of silicon-on-insulator (SOI) WPDs. We find that WPDs exhibit a noiseless port, with important implications for receiving systems and absorption-based quantum state transformations. At the same time, the thermal signals exiting noisy ports exhibit nontrivial correlations, opening the possibility for noise cancellation. We analyze passive and active networks containing WPDs showing how such nontrivial correlations can prevent the amplification of the thermal noise introduced by WPDs while benefiting from their isolation capabilities. Using this insight, we propose a modified ring-resonator amplifier that improves by *N* times the SNR in comparison with conventional traveling wave and ring-resonator amplifiers, with *N* being the number of inputs/outputs of the WPD. We believe that our results represent an important step forward in the implementation of SOI-WPDs and their integration in complex photonic networks, particularly for mid-IR and quantum photonics applications.

## Introduction

Wilkinson power dividers (WPDs) are lossy reciprocal devices with a 1 × *N* port configuration, characterized for having all their *N* + 1 ports matched (no reflections) and having *N* mutually isolated ports.^1,2^ WPDs provide functionalities of power division/combination^1,2^ as well as coherent perfect absorption (CPA).^3−5^ The lossy character of WPDs plays a fundamental role on their mode of operation, since their matching and isolation properties are ultimately enabled by the dissipation of unwanted signals in resistor elements.^1,2^ WPDs are widely used at RF and microwave frequencies for communication and sensing applications, being a common component of transceivers, transistors, modulators, and phased arrays.^6−10^ Due to their ability to cover multiple frequency bands, they have been deployed in 5G and satellite communication infrastructures.^11−14^ In addition, WPDs have been used in networks of amplifiers,^15,16^ using paralleled configurations to increase their dynamical range. Recent reports also suggest that WPDs might increase the SNR of beam-forming networks.^17^

While the excellent properties of WPDs have been extensively exploited in RF and microwave technologies, similar concepts could be adapted to other technological platforms. For example, integrated photonics is a rapidly growing field with an increasingly dominant role in communication, sensing and LIDAR, computing, and quantum technologies as well as in neural networks and artificial intelligence.^18−26^ Therefore, the implementation of WPD concepts on a silicon-on-insulator (SOI) platform might have a transversal technological impact. Our group recently demonstrated that it is possible to design a WPD in a pure SOI platform by harnessing radiative losses.^3^ In contrast to conventional WPDs at RF and microwave frequencies, the proposed SOI-WPD does not require the integration of resistor materials. Instead, the isolation between adjacent ports is achieved by radiation losses, enabling an all-silicon structure with a small footprint. In general, the undesired signals are radiated upward, and they are extracted from the chip with no impact on the optical performance of the network. However, there might be densely packaged configurations, network topologies, and/or packaging environments where the radiation might affect the performance of the optical network. In those cases, implementations of WPDs based on plasmonic coherent perfect absorbers (CPAs)^27,28^ or beamsplitters with blackened ports might be preferred, as the unwanted signal is dissipated into a lossy material, avoiding any potential interference effect. In addition, it was found that WPDs grant access to CPA quantum state transformations in photonic quantum networks with a reduced footprint and a smaller number of components.^3^ These results motivate research on how to further transfer the advantages of WPDs from RF and microwave technologies to integrated photonics.

A crucial aspect in the implementation of lossy devices is accurately accounting for their noise performance.^29−32^ Following the fluctuation–dissipation theorem,^33−35^ all lossy devices necessarily support fluctuating currents that introduce thermal noise into the system. This effect is particularly relevant in networks containing elements with gain, where independent sources must be introduced for the active and lossy elements^29,30^ and where thermal noise signals might be amplified. In this work, we investigate the noise performance of passive and active networks containing WPDs, in view of their implementation in integrated photonic platforms. Our analysis aims to clarify the physical origin of the thermal noise in SOI-WPDs and the properties of the generated noise signals. Although the impact of thermal radiation is low in the laboratory conditions of the SOI wavelength band, this study is relevant as the environmental and temperature conditions worsen in real applications, when the intensity of the input signal decreases to the minimum, as in quantum devices, or when other wavelength ranges are explored, such as midrange infrared for sensing applications.^20,36^ We believe that our analysis also provides a clearer picture of known configurations of WPDs and recent reports of the enhancement of the SNR in receiving antenna arrays.^17^ Moreover, our analysis extends to network architectures that are of much interest to integrated photonic systems, including ring-resonator amplifiers with an improvement in the SNR directly proportional to the number of WPD-ports.

## Results and Discussion

### Thermal Emission from Wilkinson Power Dividers

**Figure 1:**
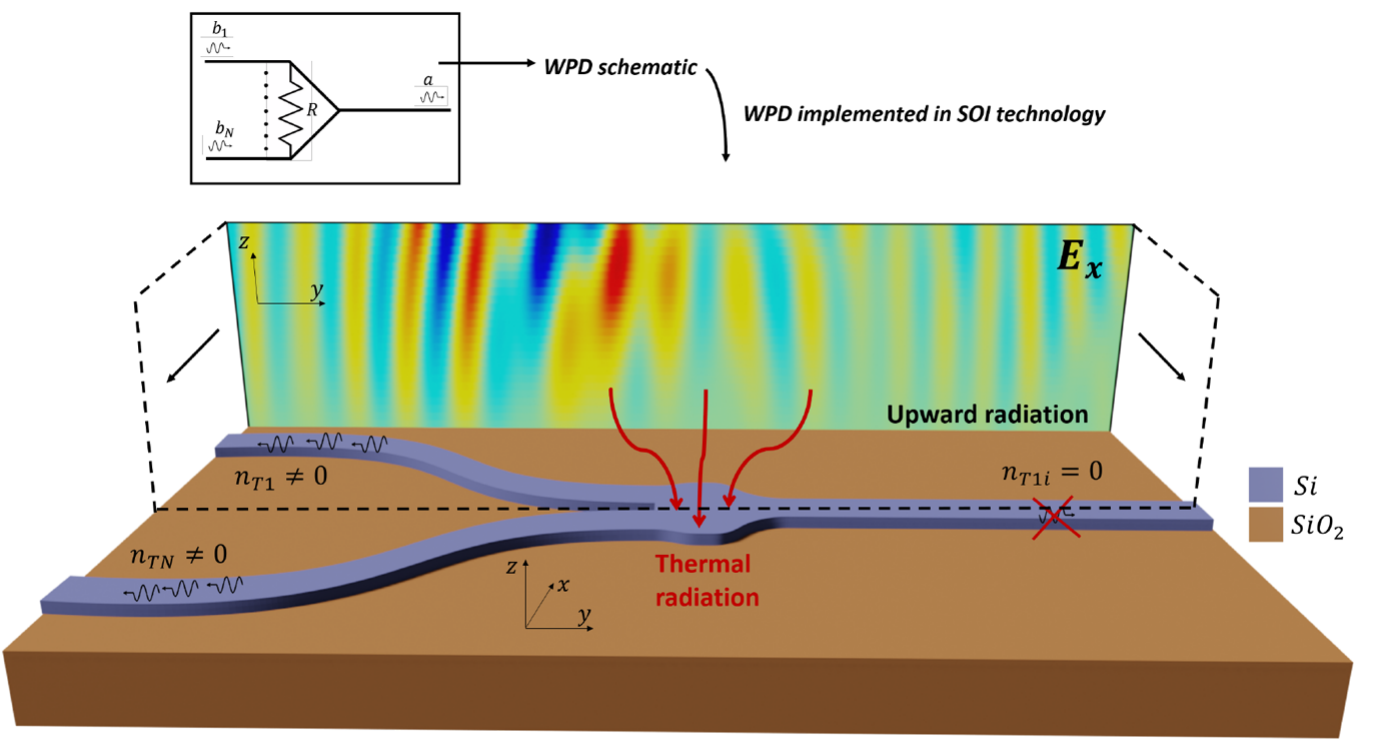
Schematic depiction of thermal noise emission in integrated photonics Wilkinson power dividers (WPDs). Simulations correspond to the real part of the *E*_*x*_ component. Inset: Circuit equivalent of a WPD.

We start by analyzing the thermal emission from a SOI-WPD,^3^ whose equivalent circuit and implementation based on a Y-branch with an engineered lateral profile are depicted in Figure 1. The scattering matrix and additional information on the designed device are reported in Supporting Information Section 1. Thermal emission is a physical phenomenon in which a device generates outgoing thermal radiation due to its nonzero temperature. Beyond the response of a device as a thermal emitter, understanding thermal emission provides an intuitive picture of the noise signals added to the system by a lossy device and the correlations between them.

Interestingly, the physical origin of thermal noise in SOI-WPD is very different from that of conventional WPDs. In RF and microwave technologies, thermal noise generation is associated with the presence of resistor elements, which dissipate power and whose fluctuating currents radiate noise into the system.^37^ Similarly, nanophotonic thermal emitters are fundamentally driven by the fluctuation–dissipation theorem,^34^ where local dissipation is intrinsically linked to the sources of thermal radiation. However, the equivalent of a WPD in integrated optics is a device with no significant dissipation elements in the sense of material loss. On the contrary, the losses enabling the performance as a WPD stem from the radiation of the propagating mode. Despite this fact, it must be remarked that all lossy devices introduce noise into the system. In this case, the thermal emission introduced by the SOI-WPD is not locally generated, but it is coupled from an external environment at temperature *T* through radiation channels (see Figure 1). Thus, the radiation can be understood as a continuum of ports in the device that (i) provide isolation capabilities and (ii) introduce the thermal noise into the system. Figure 1 shows the implementation of the WPD in SOI technology and the radiation of the real part *E*_*x*_ component in the top and side view that was performed in the *x*-coordinate corresponding to the middle of the WPD, as indicated with dashed lines. The simulation was carried out at 1550 nm and in the isolated configuration, i.e., when the device is excited by the top-left port. Simulations were carried out with a full-wave numerical solver.^38^

Following the scattering formalism,^29,30^ the thermal power emission can be calculated by the noise correlation matrix
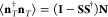
1where < > defines the ensemble average, so that the matrix  represents the correlation between the thermal noise signals exiting the device. In particular, **n**_*T*_ = [*n*_*T*1*i*_, *n*_*T*1_, ..., *n*_*TN*_] is a row vector containing the thermal noise signal exiting each port, and **N** = *N*_*T*_**I** with  is a noise matrix corresponding to blackbody radiation. In the low-frequency limit, *ℏω*/*k*_*B*_*T* ≪ 1, *N*_*T*_ converges to the *k*_*B*_*T* formalism employed in the microwave literature. **S** is the scattering matrix describing the linear response of the device that, for a balanced 1 × *N*-port WPD, is given by
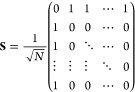
2so that the noise correlation matrix eq 1 can be explicitly written as
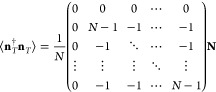
3

Notice that the 1*i* port (see Figure 1) is the port that corresponds to the first row and column in the above matrices, and in this manner, such matrices are defined as (*N* + 1) × (*N* + 1) square matrices.

The *diagonal elements* of the noise correlation matrix in eq 3 describe the thermal noise power emitted through each port. This result is consistent with the prediction of the thermal emission via Kirchhoff’s principle,^39,40^ which states that the thermal emissivity of a port is equal to its absorptivity: . We can observe from eq 3 that the thermal noise exiting the *N* ports of the device is the same, and it equals (*N* – 1)/*N* times blackbody radiation. As anticipated, lossy devices introduce noise into the system, which must taken into account in the design procedure. At the same time, it is found from eq 3 that the thermal radiation exiting the *n* = 1 port (that corresponds with the 1*i* port) is zero. The fact that WPDs have a “noiseless port” has important technological implications. For instance, this result shows that when the WPD works as a power combiner (e.g., in a receiving antenna array), it does not add noise into the system. In such a configuration, the system can benefit from the port isolation capabilities provided by a WPD, without paying the price of adding noise. Similarly, all CPA quantum state transformations discussed in ref (3) operate in a power combining configuration. Thus, our analysis concludes that such a WPD configuration empowers a variety of absorption-based quantum state transformations^41−47^ while protecting them from the detrimental impact that the thermal noise produced in lossy devices could have in few-photon light states.

Next, the *off-diagonal elements* represent the correlations among the thermal signals exiting through different ports. As we can observe, such elements are different from zero, and a WPD shows a high degree of correlation between the thermal noise on different ports. Specifically, it can be concluded from eq 3 that the diagonal elements perfectly compensate the off-diagonal ones. In other words, the noise correlation matrix has the interesting property that, for all columns and rows, the sum of their elements is exactly zero, i.e.,
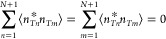
4

**Figure 2:**
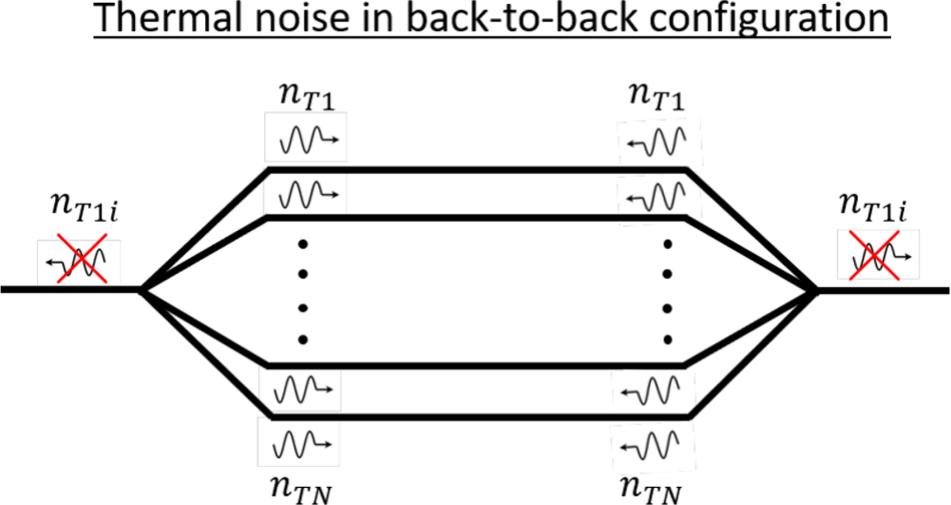
Schematic depiction of the thermal noise cancellation in a back-to-back configuration of two Wilkinson power dividers.

Eq 4 opens the possibility for noise cancellation effects when the signals are properly combined. In other words, due to the correlation of the thermal signals, we can cancel out the noise added by the system, with a profound impact on the noise performance of the system. In addition, we note that it is crucial to account for the correlation between the noise signals to correctly predict the noise response. For instance, consider a back-to-back configuration in which two WPDs first divide and subsequently combine a signal (see Figure 2). Since such a device is perfectly transparent and effectively lossless, it should not generate any noise. At the same time, it is composed by two lossy devices, which individually generate noise. It is only by accounting for the correlation between the noise signals that one can correctly predict that the final thermal noise emission of the system cancels out. For the convenience of the reader, a tutorial step by step demonstration of this effect is reported in Supporting Information, Section 1. In general, the property shown in eq 4 suggests that the thermal noise can be canceled and/or minimized with the proper design of the network architecture. That is, a better noise performance can be achieved by harnessing nontrivial correlations between the thermal noise signals.

### Amplification Systems with Wilkinson Power Dividers

**Figure 3:**
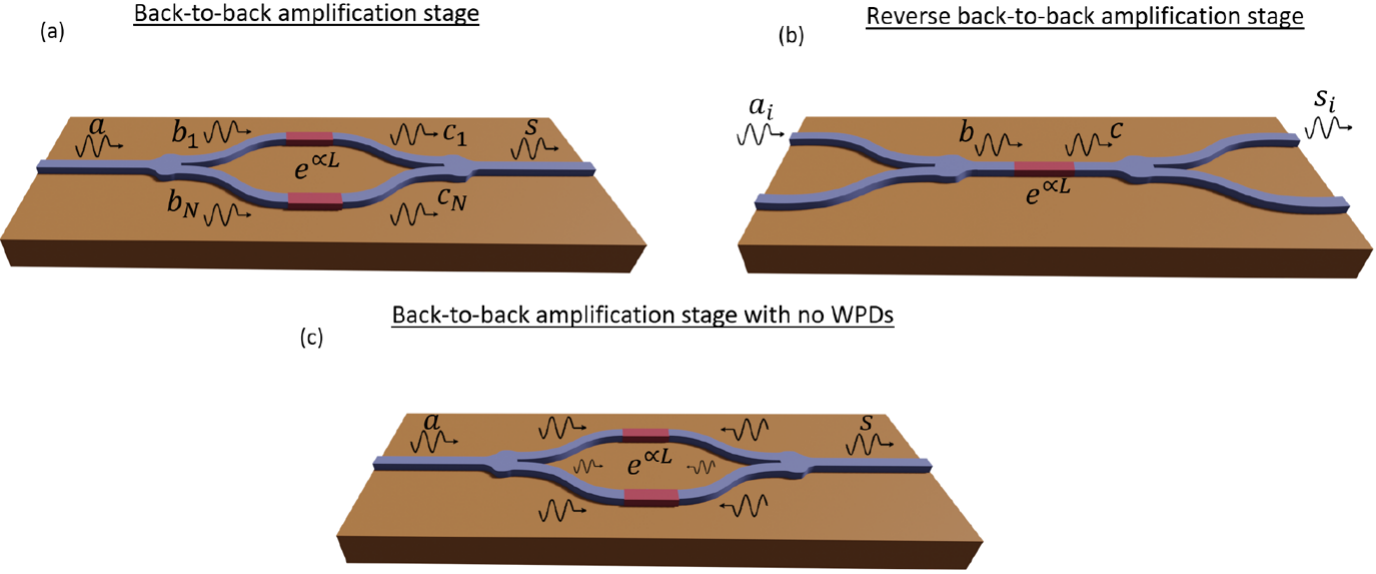
Sketch of different configurations based on the combination of Wilkinson power dividers and amplifiers, (a) *back-to-back amplification stage* and (b) *reverse back-to-back amplification stage*. (c) Sketch of the *back-to-back amplification stage* but using a different divider.

Having addressed the noise performance of a single WPD, in this section, we explore more advanced configurations containing the combination of WPDs and active devices, modeled as traveling wave amplifiers (TWAs). We will model the active units as TWAs, which could be implemented in practice, for instance, by using erbium-doped waveguides.^48^ For the sake of completeness, the noise performance analysis of a TWA is detailed in Supporting Information, Section 2. Specifically, we study the two configurations depicted in Figure 3. On the one hand, the first configuration is a *back-to-back amplification stage* (see Figure 3(a)), i.e., a two-port device (single input/single output), and it has as many amplifiers as output ports the WPD has. Such a scheme has been proposed in the RF technology, normally named the paralleled amplification stage.^15,16^ As we will demonstrate, this configuration does not improve the gain or the SNR as compared to a single amplifier. However, the use of paralleled amplifiers increases the dynamical range of the stage. On the other hand, we call the second configuration a *reverse back-to-back amplification stage* (see Figure 3(b)). This configuration consists of a 2*N*-port device, where the *N* input signals are combined with a 1 × *N*-port WPD before entering a single amplification stage. Then, the resulting signal is divided with a subsequent WPD. As we will show, this configuration provides an advantageous SNR.

The noise performance of both configurations can be analyzed with the same scattering formalism, taking into account that independent noise sources are required to model gain and loss. For the *back-to-back amplification* configuration, the signals after the first WPD are  (*n* = 1, ..., *N*), and the signals after the amplifiers are
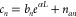
5where *n*_*an*_ is the noise signal from the *n*^th^ amplifier, α is the amplification factor, and *L* is the length of the TWA. Notice that all amplifiers are assumed with the same level of gain, but their noise signals are uncorrelated: . Finally, the signal at the output of the amplifying stage is then given by
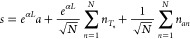
6

With this information, the signal power, total power, and SNR at the output of the amplification stage are given by
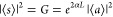
7
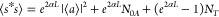
8
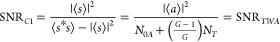
9where *G* is the gain of the system. In deriving the equations above, we made use of the fact that the noise from the WPDs and the amplifiers are uncorrelated: . In addition,  refers to the noise power of the input signal.

It can be concluded from eqs 7–9) that the back-to-back amplification provides the same gain and SNR than a single amplifier (see Section 2 in Supporting Information), despite using two 1 × *N*-port WPDs and *N* amplifiers. Again, we reemphasize that, with the use of the proper design, lossy devices do not necessarily lead to a penalization in terms of SNR. That is, although the noise signals appear to contribute and be amplified in eq 6, their contribution to the total noise power cancels out due to the nontrivial correlations reported in eq 3. Thus, the output power noise in eqs 8 and 9 only contains contributions from the input signal and the amplifier.

We emphasize that if we had used a different divider instead of a WPD, for instance, a Y-branch with different features in integrated optics, we would have had reflections, and the branches would not be isolated. Thus, the reflected signals would conform feedback channels between the two WPDs, and, therefore, the noise from the amplifier would be amplified, and the SNR will decrease (see Figure 3(c)). This effect illustrates the beneficial aspects of using WPD on SOI platforms, and the need to accurately address its noise performance. We note that while isolation could be even more strongly enforced with nonreciprocal components,^49^ these are typically bulky, lossy, and/or active devices at optical frequencies so that achieving port-to-port isolation with a reciprocal device poses several technological advantages.

For the reverse back-to-back configuration, a similar analysis of the input–output relations shows that the output signals are given by
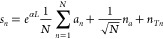
10

We assume that all input signals have the same signal component , and the same noise level, but uncorrelated noise signals, . In other words, we assume that all input ports are driven by independent sources with the same properties. Then, the signal power, total power, and signal-to-noise ratio on each of the outputs of the amplification stage are given by
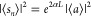
11
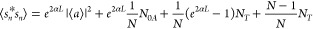
12
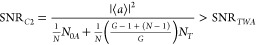
13

According to this configuration, we can conclude that the SNR is improved by a factor of *N*, i.e., defined by the 1 × *N*-port WPD. The reason for this enhancement is twofold: First, the device is driven by *N* independent sources with similar properties but uncorrelated noises. Then, the first WPD acts as a combiner, performing an average operation that improves the SNR of the input signal by a factor of *N*, as it can be appreciated by the  noise term in the denominator of eq 13. Second, since the stage uses a single amplifier, the output WPD divides the noise generated by the amplifier, resulting in a 1/*N* reduction of the noise power, as it can be appreciated from the  noise term in the denominator of eq 13. At the same time, the main drawback of this configuration is that, since it uses a single amplifier, the saturation limit will be achieved faster. For this reason, the configuration is beneficial for amplifying weak sources.

We note that, in this configuration, the presence of the last WPD results in an additional thermal noise element. This effect is evidenced by the presence of an overall *e*^2*αL*^ noise factor instead of a *e*^2*αL*^ – 1 factor in the denominator of eq 13. While this effect could be avoided by using a lossless power divider instead of a WPD, we would be losing the isolation capabilities provided by a WPD, which is an essential factor in more complex networks, as we will observe in the following section.

Interestingly, our analysis concludes that the gain in both configurations is the same. In other words, for these two configurations, the gain is independent of the WPDs and arrangement of the amplifiers. At the same time, our analysis shows that both configurations have a different noise performance, highlighting the need to account for this aspect in the design at the network level. Thus, we take advantage of the thermal radiation analysis performed in the previous section improving the SNR (as in the reverse back-to-back configuration) or maintaining the gain level but improving the dynamical range avoiding the penalization in the SNR (as in the back-to-back configuration). Next, we apply the knowledge acquired in the analysis of these two configurations to more complex network architectures.

### Wilkinson Power Dividers in Feedback Systems

In this section, we address the implementation of WPDs and gain elements in resonant systems containing feedback loops. These devices can be exploited for laser applications or high-quality amplifiers, combining the advantages that were discussed in the last section with the features provided by feedback. We will show how they provide compact and resonantly enhanced amplification while reducing the thermal noise generation with respect to traveling wave and ring-resonator amplifiers.

**Figure 4:**
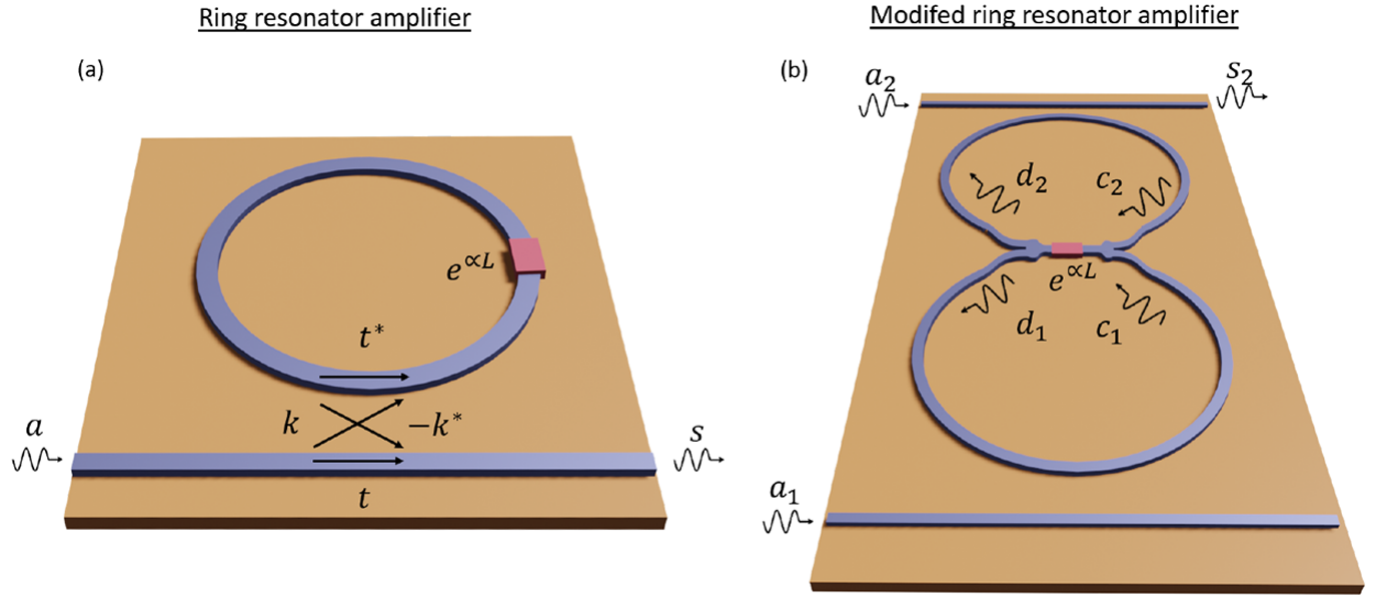
(a) Sketch of a ring-resonator amplifier and (b) the modified ring resonator with two 1 × 2 WPDs and the amplifier located between the WPDs.

Herein, we will use the ring resonator as a case study of a feedback system (see Figure 4(a)), as it has been one of the driving forces for the implementation of resonant integrated photonic devices. Ring resonators have been widely used in integrated optics by different configurations, as strip or slotted-waveguide ring resonators, periodic subwavelength gratings, based on photonic crystal and by cascading ring resonators.^50−54^ Here, we take advantage of the insight from the two configurations studied in the previous section to show how WPDs can benefit feedback systems, for example by reducing the thermal noise generated by ring-resonator amplifiers.

A ring resonator can be understood as the combination of a directional coupler and a feedback loop.^55−57^ First, the directional coupler is modeled by the input–output relations
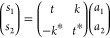
14where *k* and *t* are the coupling and transmission coefficients, respectively. Both parameters are related by  for a lossless directional coupler. Next, the active feedback loop, which defines the back connection and the amplification condition of the system, is characterized by the input–output relation
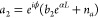
15where  is the phase accumulated after one loop in the ring, *δω* is the frequency detuning between the working wavelength and the resonance wavelength of the resonator, *c* is the speed of light in a vacuum, and *L* is the length of the ring. Losses in the ring resonator could be included in our theoretical model by adding an additional thermal noise term in eq 15.

By combining eqs 14 and 15, we find that the gain and the SNR of a ring-resonator amplifier at resonance (ϕ = 2π) are given by
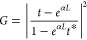
16
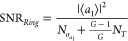
17

Next, by combining the WPD-based configurations studied in the last section and the ring resonator, we propose a modified ring-resonator amplifier with a reduced thermal noise generation. Such a device is depicted in Figure 4(b). The design of the device is justified by the two configurations examined in the previous section. First, we use a reversed back-to-back configuration to reduce the noise of the amplification stage. Second, we perform a back-to-back configuration when closing the feedback loops, thus preventing the amplification of the thermal noise introduced by the WPDs. The design depicted in Figure 4(b) is based on a 1 × (*N* = 2)-port WPD. However, the theory derived below is kept general for the 1 × *N*-port WPD. While the implementation of this concept might be challenging for a number of channels larger than 2 (*N* > 2), we keep the general *N*-port theory to illustrate the theoretical potential of the idea and in case it could be implemented in a different technological platform.

We start by defining the signals at the left *d*_*n*_ and right *c*_*n*_ of the amplification system as well as the input *a*_*n*_ and output *s*_*n*_ signals. Then, these signals are related via input–output relations as follows
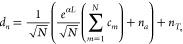
18
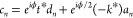
19
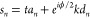
20where the input *a*_*n*_ is the combination of the signal *a*_*s*_ and noise distribution *n*_*n*_ as *a*_*n*_ = *a*_*s*_ + *n*_*n*_. This recursive system is solved by combining eqs 18 and 19 and finally introducing the result into eq 20, so that we obtain the output signal *s*_*n*_ as a function of input signals and noise sources.

To combine such equations, we have to consider two relevant factors to simplify the calculations. First, the signals at the WPD output ports can be expressed as the addition of signal and system noise parts, where the system noise encompasses the noise from the amplifier and the noise from the WPDs. Second, the signal part and the amplifier noise are the same in all the ports, but the noise signals from the WPD are not. Thus, we write , which relates the *n*^th^ port signal to the signal in the *m* port. Consequently, by adding the signals from all ports, we find . Identical performance can be carried out with the input signal (*a*_*m*_ = *a*_*n*_ – *n*_*n*_ + *n*_*m*_); thus, . Physically, this assumption on the input signals implies that they are generated by similar but independent sources. Thus, input signals present the same amplitude, but the noise distribution is different and uncorrelated among them. Finally, the output signal can be explicitly written as
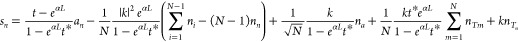
21

Consequently, the gain and SNR at the output of the amplifier stage are given by
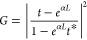
22
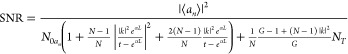
23

Finally, from the above equation, one can be observe that for high gains (when *e*^*αL*^ tends to , see eq 22), the SNR can be written as
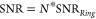
24

In fact, as we will show next with numerical calculations, this theoretical limit is rapidly reached even with moderate/low gains. Thus, we have demonstrated that by using WPDs in ring-resonator-based systems, the SNR at the output of the amplifier can be improved by a factor of *N*. See Supporting Information for more detail.

In order to understand the gain and noise performance of the system, we analyze the physical meaning of eq 21 term by term. The first term is directly proportional to the input signal: , and it corresponds to the gain of the system, as explicitly stated in eq 22. Interestingly, we find the gain identical to the one of a conventional ring-resonator amplifier,^58^ given by eq 16. This equivalence is justified because both resonators work at the wavelength resonance, and for this reason, the topology of the resonator, i.e., the shape and number of rings for instance, has no impact on the gain. In addition, the gain depends on the active elements, and in both cases, this is provided by one TWA, because the WPD is a passive device. Thus, we find that although we include a 1 × *N*-port WPD and *N* ring resonators, we still have, in essence, a resonator with only one TWA, which is the same scenario as a conventional ring-resonator amplifier. On the other hand, we demonstrated in the previous section comparing the *back-to-back amplification stage* and the *reverse back-to-back amplification stage* that combining signals first and then amplifying and dividing them does not modify the gain. For this reason, the signal processing by combining two or more input signals (as in the modified ring resonator in Figure 4(b)) does not change the gain in the systems either.

According to eq 21, there is a term based on the noise distribution of the input signals: . This term appears because the input signals come from different sources, and thus, the noise background differs. As we can observe, these noises are amplified by the TWA and then, conducted to the output, mixed, and split by  the noise from all the input signals in the WPD stage.

Aside from the signal noise, the internal noise performance also deviates from that of a ring-resonator amplifier. The second term describes the noise introduced by the active element, . In comparison with the ring resonator with no WPD, the gain amplifier noise is divided by a factor of *N*, the number of ports and rings. This can be explained by the fact that we are using only one amplifier before the division of the signal, and for that reason, the noise from such a TWA is divided according to the number of ports. Thus, similar to the reverse back-to-back configuration, the noise introduced by the amplifier is reduced. Our analysis is not only restricted to cancel the thermal noise, as we demonstrate the impact on the amplifier’s noise as well, but also may be relevant progress on integrated photonics, where the development of low-noise amplifiers is a very active field of research.^59−61^ In addition, while have used a simple TWA model, the same conclusions would hold for more realistic amplifiers with additional noise sources (see Supporting Information, Section 2).

The third term is the thermal emission noise, associated with the noise sources . Eq 21 shows that the thermal noise signals introduced by the WPD are amplified as they enter the feedback loop. However, despite the amplification of the individual signals, their overall contribution to the noise power is zero due to the nontrivial correlations among them. In particular, the two WPDs in the configuration form a back-to-back stage when closing the feedback loop. Thus, the thermal noise that remains in the system is canceled as described in previous sections. Mathematically, one can observe that, when computing the output power , the term: , cancels out due to the correlations described in eq 4.

The final term is proportional to *n*_*Tn*_ alone, and it corresponds to the thermal noise that is directly leaked outside the system. This term appears because not all the thermal emission is fed back into the loop, but it is also partially coupled outside with coupling factor *k*. Thus, the only net thermal noise contribution from the WPD is not amplified by the active elements.

Therefore, one of the most relevant features of this configuration is that it prevents the amplification of the thermal noise generated by the WPDs. In other words, it is possible to combine lossy and active elements in feedback systems while preventing the amplification of the thermal noise, which would have a very detrimental impact on the SNR.

At the same time, one can benefit from the capabilities offered by lossy devices. For example, thanks to the isolation properties of WPDs, there is a guarantee that there are no backpropagating signals in the feedback loop. Thus, it is possible to avoid backpropagating noise amplification that would increase the noise in the system. In our configuration, the backpropagating power is simply , corresponding to the unamplified thermal noise signal that couples to the exit (that corresponds with the input in the top waveguide in Figure 4(b)).

**Figure 5:**
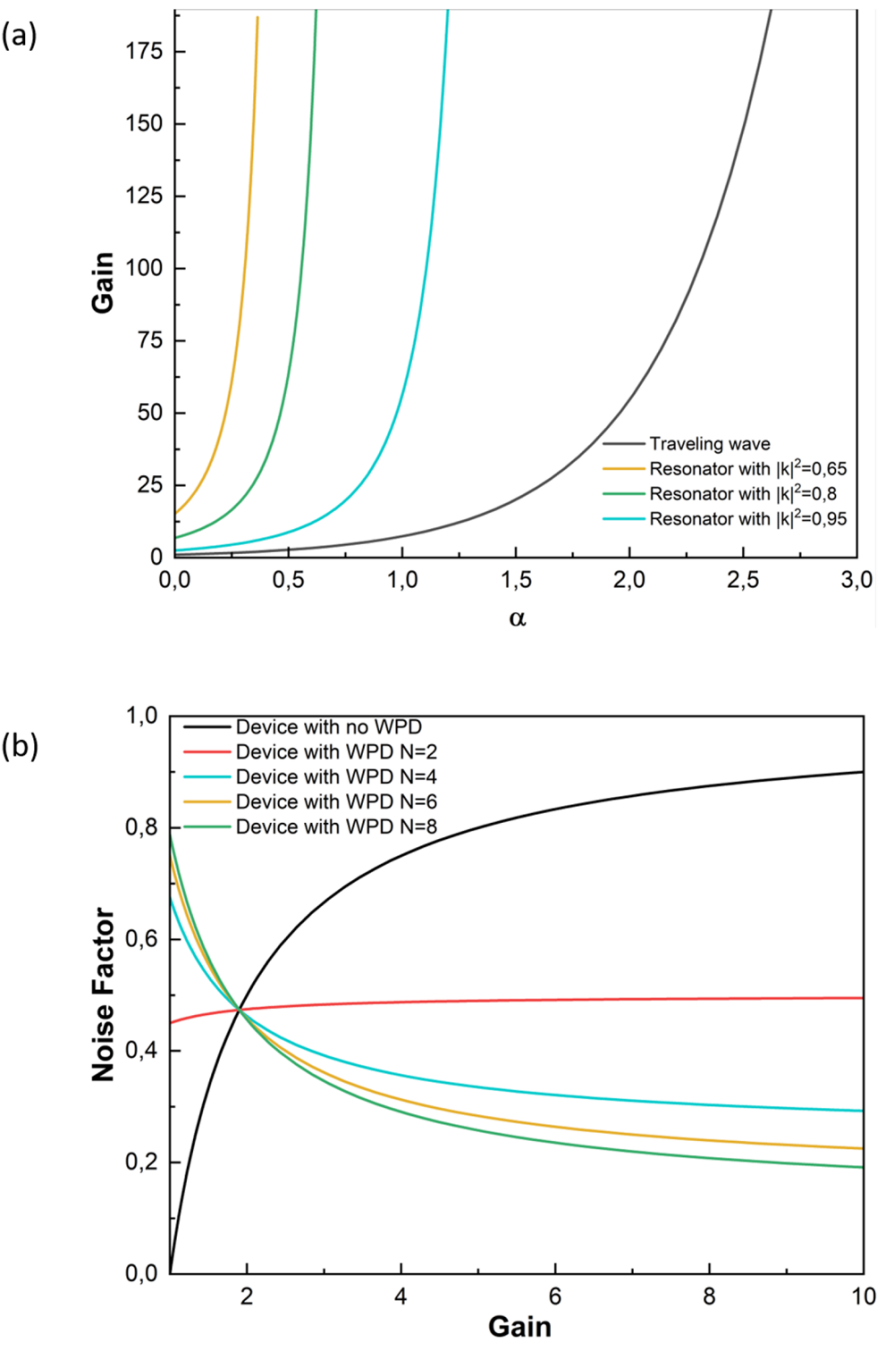
(a) Evolution of the gain in the system based on the combination of WPDs, ring resonators, and TWAs for different couplings |*k*|^2^, compared with the one provided by only the TWA, and (b) the impact of the noise factor for different 1 × *N*-port WPDs for a |*k*|^2^ = 0.95.

As we can observe from these results, we can reduce the total noise associated with the system (both from the signal and from the thermal emission) by a factor of *N*. Thus, we improve the SNR by *N*, at the cost of increasing the elements in the final device (in terms of ports in the WPDs and resonance rings). This is an interesting result that provides us the opportunity to develop high-quality amplifiers that improve the SNR in comparison to conventional amplifiers. This can be observed in the results reported on Figure 5. First, Figure 5(a) shows the gain of the TWA and the modified ring resonators with different coupling factors as a function of the gain coefficient α. Whereas the gain in the TWA increases exponentially, the gain for ring-resonator configurations diverges at the points for which t*e^*αL*^ = 1. The result illustrates how ring resonators allow for large gains in a compact device. Naturally, a diverging gain is unphysical, and the growing trend will stop when saturation and other nonlinear effects kick in. Therefore, the analysis has to be restricted for practical gains within the t*e^*αL*^ < 1 region. Taking this into account, we compare the noise performance of the resonators for the same level of gain and for a |*k*|^2^ = 0.95, as shown in Figure 5(b). Following eq 23, it can be concluded that, for sufficiently large gains, the noise of the system can be reduced by a factor of *N*. In fact, even for moderate and low gains (*G* > 2) the noise performance of the amplifier already exceeds that of a conventional ring-resonator amplifier. The most simple case, *N* = 2, whose practical implementation is depicted in Figure 4(b), would provide an improvement as twice as high.

**Figure 6:**
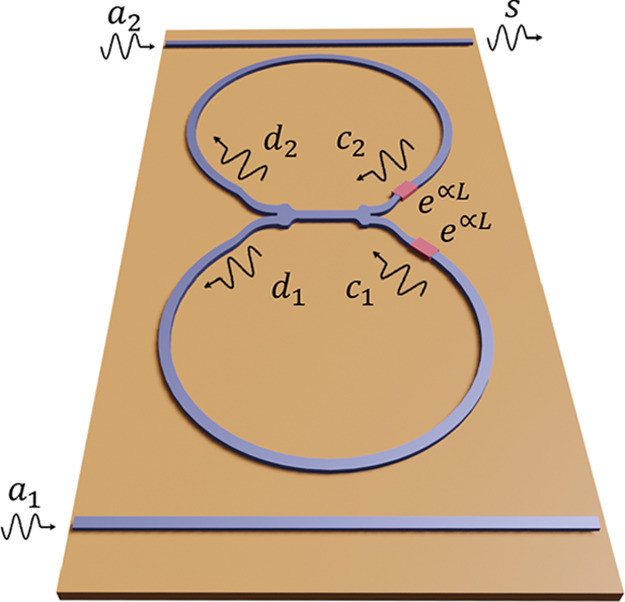
Modified ring resonator with two 1 × 2 WPDs and the amplifier located in the rings.

Therefore, our analysis concludes that the use of WPDs in feedback configurations such as ring resonators allows for amplification stages that provide both large gain in a compact system as well as an enhanced SNR. Due to the complex interplay between the loss and gain noise signals, the analysis of the noise performance is essential in such configurations. We anticipate that many other designs would be possible, also benefiting from the isolation capabilities of WPDs while maintaining the thermal noise associated with a lossy device under control. For example, the configuration depicted in Figure 6 provides exactly the same performance in terms of gain and SNR, as shown in Supporting Information, Section 3. One can envision the design of a more complex network with a large number and/or different classes of active elements.

## Conclusions

We have reported a theoretical study of the thermal emission from WPDs and the noise performance of photonic networks containing WPDs and gain elements, in view of their application in integrated photonic systems. Here, we clarify both the physical origin and the properties of the noise generated by integrated photonics WPDs, including nontrivial correlations between the noise signals exiting different ports. Our results highlight how the noise generated by lossy devices has nontrivial properties that can be exploited in nanophotonic engineering. First, WPDs have a noiseless port so that they do not add noise into the system when acting as a power combiner. This effect has important implications for receiving communication and/or LIDAR systems and for absorption-based quantum state transformations. Second, the nontrivial correlations between the noise signals lead to a cancellation of the overall thermal noise in specific configurations. Based on this property, we designed a modified ring-resonator resonant amplifier that exhibits resonantly enhanced gain in a compact device and an improvement in the SNR as compared to conventional ring-resonator and traveling wave amplifiers. We believe that our results open a new direction in the design of integrated photonic amplifiers. We also expect that similar ideas could be exploited in the design of more complex photonic networks and/or integrated light sources.

In general, our results demonstrate that the nanophotonic design of noise provides access to the extra capabilities added by lossy devices (e.g., port isolation and absorption-based quantum state transformations) while mitigating the detrimental impact of the thermal noise generated by them. We believe that our results represent an important step forward in the implementation of lossy devices, such as WPDS, in integrated photonic networks.

## Methods

Numerical results in Figure 1 were computed by using Ansys Lumerical as a full-wave numerical solver.^38^ Numerical results in Figure 5 were computed by direct evaluation in Mathematica.

## Supporting Information Available

Additional information on the SOI-WPD design and the TWA model applied, the theoretical approach of the system proposed, and a deep explanation of thermal radiation in back-to-back WPDs (PDF)
