# Supplementary material for: Noise Cancellation Effects in Integrated Photonics with Wilkinson Power Dividers
Source: ACS Photonics. 2023 Apr 12;10(5):1240–9. doi: 10.1021/acsphotonics.2c01675 (PMC10197118; doi:10.1021/acsphotonics.2c01675)
Supplement: Supplementary file 1 — ph2c01675_si_001.pdf [file ph2c01675_si_001.pdf]

Supporting Information: Noise cancellation effects in integrated photonics with Wilkinson power dividers

Angel Ortega-Gomez,* Osmery Hernández, Douglas Oña, Carlos Biurrun-Quel, Carlos del Río, and Iñigo Liberal*

E-mail: angel.ortega@unavarra.es; inigo.liberal@unavarra.es

Section 1: Wilkinson Power Divider in SOI

The integration of WPD in SOI is performed by a Y-branch with an engineered lateral profile, as it is explained in the main text. To show how the response of this device matches that of a WPD, we report the scattering matrix of both, the ideal WPD (Fig. S1(a)) and the SOI-WPD designed (Fig. S1(b)).

Section 2: Thermal radiation in back-to-back WPDs

In this section we analyze the noise performance of a back-to-back configuration of two $1 \times N$-port WPDs as shown in Figure 2. To this end, we will evaluate the SNR in the c-port.

First, applying the input-output relations for a WPD we have that the signals after the first WPD are given by

$$b_i = \frac{1}{\sqrt{N}} a + n_{Ti} \tag{1}$$

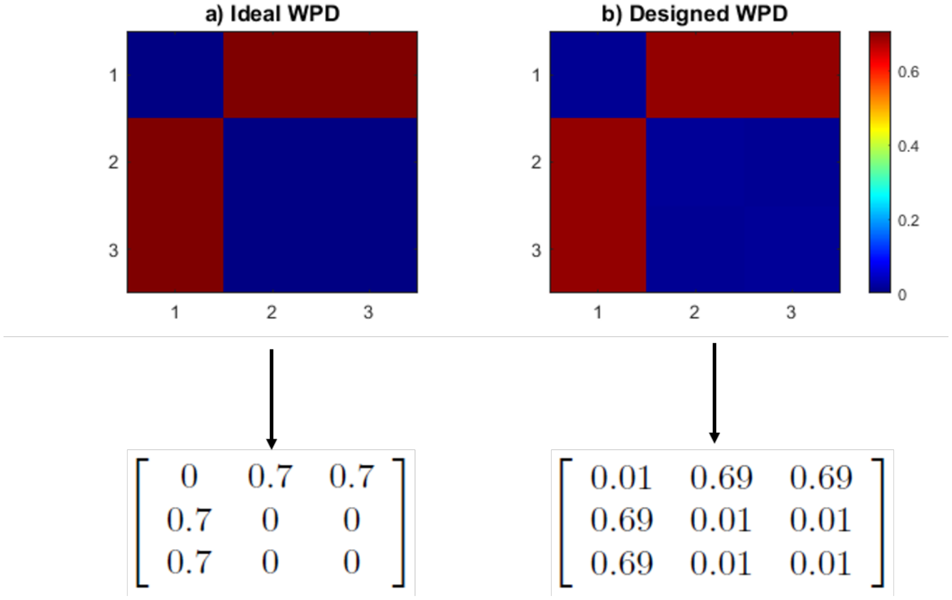

Figure S1 –Scattering matrix of the ideal WPD (a) and designed SOI-WPD (b).

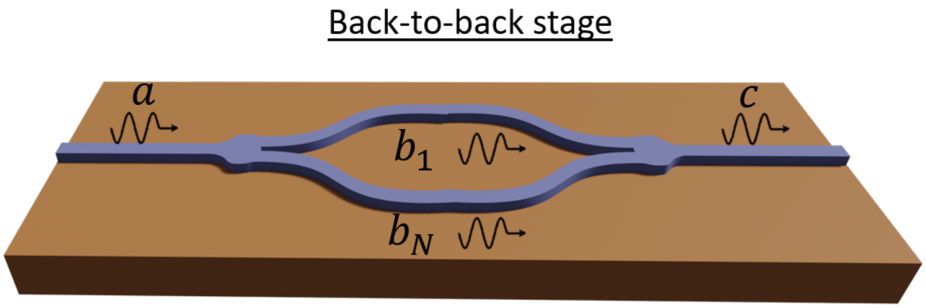

Figure S2 –Sketch of the integrated back-to-back configuration.

and the signal after the second WPD can be defined as:

$$c = \frac{1}{\sqrt{N}} \left(\sum_{i=1}^{N} b_i \right) = a + \frac{1}{\sqrt{N}} \left(\sum_{i=1}^{N} n_{Ti} \right) \tag{2}$$

where n_{Ti} is the thermal noise in the i port.

Next, the signal to noise ratio is given by

$$SNR_c = \frac{|\langle c \rangle|^2}{\langle c^* c \rangle - |\langle c \rangle|^2} \tag{3}$$

where

$$|\langle c \rangle|^2 = |\langle a \rangle|^2 + \frac{1}{\sqrt{N}} \left\langle \sum_{i=1}^{N} n_{Ti} \right\rangle^2 = |\langle a \rangle|^2 \tag{4}$$

$$\langle c^* c \rangle = \langle a^* a \rangle + \frac{1}{N} \left(\sum_{i,j=1}^{N} \langle n_{Ti}^* n_{Tj} \rangle \right) \tag{5}$$

where $\left\langle \sum_{i=1}^{N} n_{Ti} \right\rangle^2 = 0$, as the average of thermal noise is defined as zero. In order to obtain the previous equation, we have calculated the thermal power emission by the noise correlation matrix:

$$\left\langle \mathbf{n}_T^\dagger \mathbf{n}_T \right\rangle = (\mathbf{I} - \mathbf{S}\mathbf{S}^\dagger)\mathbf{N} = \frac{1}{N} \begin{pmatrix} 0 & 0 & 0 & \cdots & 0 \\ 0 & N-1 & -1 & \cdots & -1 \\ 0 & -1 & \ddots & \cdots & -1 \\ \vdots & \vdots & \vdots & \ddots & \vdots \\ 0 & -1 & -1 & \cdots & N-1 \end{pmatrix} N_T \tag{6}$$

Thus, we obtain that

$$\langle c^* c \rangle = \langle a^* a \rangle + N_T \frac{1}{N} \left(\sum_{i=1}^{N} (N-1) - \sum_{i=1}^{N} (\sum_{j=1}^{N-1} 1) \right) = \langle a^* a \rangle = |\langle a \rangle|^2 + N_{0a} \tag{7}$$

where N_{0a} is the noise of the input signal. We highlight that the contribution from the thermal emission to the total power cancels out $\left(\sum_{i=1}^{N} (N-1) - \sum_{i=1}^{N} (\sum_{j=1}^{N-1} 1) \right)$, where

the first term corresponds to the contribution of the diagonal elements, and the second term corresponds to the contribution of the off-diagonal elements.

Finally, the signal to noise ratio reduces to

$$SNR_c = \frac{|\langle c \rangle|^2}{\langle c^* c \rangle - |\langle c \rangle|^2} = \frac{|\langle a \rangle|^2}{N_{0a}} = SNR_a \tag{8}$$

It can be concluded from this result that the SNR at the output is the same than at the input, due to the thermal noise cancellation because of the combination of the diagonal elements and all the nontrivial correlations (off-diagonal elements).

Section 3: Travelling wave amplifier

Here we show the noise performance analysis of the TWA (see Figure S 3). The scattering matrix of this device is as follows:

$$\mathbf{S} = \begin{pmatrix} 0 & e^{\alpha L} \\ e^{\alpha L} & 0 \end{pmatrix} \tag{9}$$

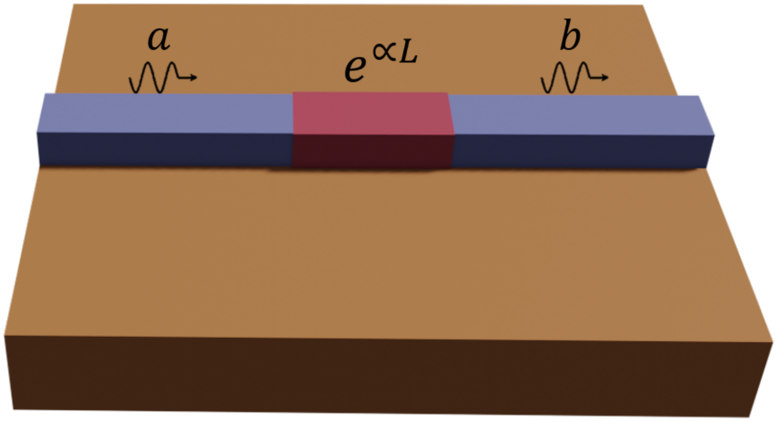

Figure S3 –Scheme of TWA implemented in a silicon-on-insulator (SOI) platform.

When $\alpha > 0$, the TWA behaves as amplifier, and when $\alpha < 0$, it behaves as a lossy device. Notice that $\alpha = 0$ refers to a neutral element with no gain and loss.

The output can be expressed as:

$$b = ae^{\alpha L} + n_a \tag{10}$$

being n_a the noise generated by the amplifier. Thus, the SNR can be calculated as:

$$|\langle b \rangle|^2 = e^{2\alpha L} |\langle a \rangle|^2 \tag{11}$$

$$\langle b^* b \rangle = e^{2\alpha L} \langle a^* a \rangle + \langle n_a^* n_a \rangle \tag{12}$$

where:

$$\left\langle \mathbf{n}_a^\dagger \mathbf{n}_a \right\rangle = (\mathbf{S}\mathbf{S}^\dagger - \mathbf{I})\mathbf{N} = \begin{pmatrix} e^{2\alpha L} - 1 & 0 \\ 0 & e^{2\alpha L} - 1 \end{pmatrix} N_T \tag{13}$$

Finally, the SNR at the output of the amplifier can be expressed as:

$$SNR_{TWA} = \frac{|\langle b \rangle|^2}{\langle b^* b \rangle - |\langle b \rangle|^2} = \frac{e^{2\alpha L} |\langle a \rangle|^2}{e^{2\alpha L} N_{0b} + \left(e^{2\alpha L} - 1\right) N_T} = \frac{|\langle a \rangle|^2}{N_{0A} + \left(\frac{G-1}{G}\right) N_T} \tag{14}$$

We note that more realistic amplifiers present additional noise signals in addition to the minimum thermal noise. In order to model this case, the amplifier's noise can be expressed as:

$$n_a = n_{aT} + n_A \tag{15}$$

where n_{aT} corresponds with the one studied previously and n_A models additional noise signals, with the following properties:

$$\langle n_{aT}^* n_A \rangle = 0 \tag{16}$$

$$\langle n_A^* n_A \rangle = r_A \mathbf{I} \tag{17}$$

$$\langle n_a^* n_a \rangle = \left(e^{2\alpha L} - 1\right) N_T \mathbf{I} + r_A \mathbf{I} \tag{18}$$

Where $r_A\mathbf{I}$ corresponds with the correlation factor of n_A and the two parts of the amplifier's noise are uncorrelated between them. Then, Eq.(14) is generalized by substituting the minimal noise $(G-1)N_T$ by $\left(e^{2\alpha L} - 1\right) N_T + r_A$.

Section 4: Wilkinson power divider (WPD) within a ring resonator network

Here, we show the theoretical analysis of the device depicted in Figure 5 on the main text for the case $N = 2$. In this configuration, the TWAs are located within the ring resonators. Therefore, the input-output equations that describe this device are the following:

$$d_2 = \frac{1}{\sqrt{2}} \left(\frac{1}{\sqrt{2}} (c_1 + c_2) \right) + n_{T_2} \tag{19}$$

$$c_1 = e^{i\phi} t^* d_1 + e^{\frac{i\phi}{2}} (-k^*) a_1 + n_{a1} \tag{20}$$

$$c_2 = e^{i\phi} t^* d_2 + e^{\frac{i\phi}{2}} (-k^*) a_2 + n_{a2} \tag{21}$$

$$s_2 = a_2 t + e^{\frac{i\phi}{2}} k d_2 \tag{22}$$

$$a_i = a_s + n_i \tag{23}$$

where a_s and n_i are the amplitude and noise distribution of the ith input signal.

Consequently, the output signal is given by

$$s_2 = \frac{t - e^{\alpha L}}{1 - e^{\alpha L} t^*} a_2 + \frac{1}{2} \frac{|k|^2 e^{\alpha L}}{1 - e^{\alpha L} t^*} (n_1 - n_2)$$

$$+\frac{1}{\sqrt{2}}\frac{k}{1-e^{\alpha L}t^*}\left(n_{a1}+n_{a2}\right)+\frac{1}{2}\frac{kt^*e^{\alpha L}}{1-e^{\alpha L}t^*}\left(n_{T1}+n_{T2}\right)+kn_{T_2} \tag{24}$$

By using Eqs.(19-24), we can calculate the SNR, being the intensity and the cross products the following:

$$|\langle s\rangle|^2=\left|\frac{t-e^{\alpha L}}{1-e^{\alpha L}t^*}\right|^2|\langle a_2\rangle|^2 \tag{25}$$

$$\begin{aligned}\langle s^*s\rangle=\left|\frac{t-e^{\alpha L}}{1-e^{\alpha L}t^*}\right|^2\langle a^*a\rangle+\frac{1}{4}\frac{|k|^4e^{2\alpha L}}{|1-e^{\alpha L}t^*|^2}\langle n_1^*n_1\rangle+\frac{1}{4}\frac{|k|^4e^{2\alpha L}}{|1-e^{\alpha L}t^*|^2}\langle n_2^*n_2\rangle+\\ \frac{1}{2}\frac{|k|^2e^{\alpha L}(t-e^{\alpha L})^*}{|1-e^{\alpha L}t^*|^2}\langle a_2^*n_2\rangle+\frac{1}{2}\frac{|k|^2e^{\alpha L}(t-e^{\alpha L})}{|1-e^{\alpha L}t^*|^2}\langle n_2^*a_2\rangle+\\ \frac{1}{4}\frac{|t|^2|k|^2e^{2\alpha L}}{|1-e^{\alpha L}t^*|^2}\left(\langle n_{T1}^*n_{T1}\rangle+\langle n_{T2}^*n_{T2}\rangle+\langle n_{T1}^*n_{T2}\rangle+\langle n_{T2}^*n_{T1}\rangle\right)+\\ \frac{1}{2}|k|^2\langle n_2^*n_2\rangle+\frac{1}{4}\frac{|k|^2(e^{2\alpha L}-1)}{|1-e^{\alpha L}t^*|^2}\left(\langle n_{a1}^*n_{a1}\rangle+\langle n_{a2}^*n_{a2}\rangle\right)\end{aligned} \tag{26}$$

Assuming the following statements: $\langle n_{T1}^*n_{T1}\rangle+\langle n_{T2}^*n_{T2}\rangle+\langle n_{T1}^*n_{T2}\rangle+\langle n_{T2}^*n_{T1}\rangle=0$,$\langle a^*a\rangle=|\langle a_2\rangle|^2+\langle a_2^*a_2\rangle$, $\langle n_i^*n_j\rangle=0$, $\langle n_2^*n_2\rangle=\langle n_1^*n_1\rangle=\langle a_2^*n_2\rangle=\langle n_2^*a_2\rangle=N_0$; the gain and the SNR of the amplifying state can be written as

$$G=\left|\frac{t-e^{\alpha L}}{1-e^{\alpha L}t^*}\right|^2 \tag{27}$$

$$SNR=\frac{|\langle a_n\rangle|^2}{N_0\left(1+\frac{N-1}{N}|\frac{|k|^2e^{\alpha L}}{t-e^{\alpha L}}|^2+\frac{2(N-1)}{N}\frac{|k|^2e^{\alpha L}}{t-e^{\alpha L}}\right)+\frac{1}{N}\frac{G-1+(N-1)|k|^2}{G}N_T} \tag{28}$$

That is the same result that the device analyzed in the main text with only one amplifier but located between the two WPDs.

Now, we can approach the SNR for high gain, considering that the gain tends to infinite when $e^{\alpha L}=\frac{1}{t^*}$. On the one hand, according with the term related to the thermal noise when $G\rightarrow\infty$ we have:

$$\frac{1}{N}\frac{G-1+(N-1)|k|^2}{G}=\frac{1}{N} \tag{29}$$

On the other hand, the term related to the signal noise can be approached as:

$$1+\frac{N-1}{N}\left|\frac{|k|^2e^{\alpha L}}{t-e^{\alpha L}}\right|^2+\frac{2(N-1)}{N}\frac{|k|^2e^{\alpha L}}{t-e^{\alpha L}}=1+\frac{N-1}{N}-\frac{2(N-1)}{N}=\frac{1}{N} \tag{30}$$

Thus, the SNR can be written for high gain as

$$SNR = N * SNR_{Ring} \tag{31}$$

Nevertheless, as we demonstrate in Figure 5 (main text), with a gain greater that 2 (in lineal terms), the improvement in the noise is significant.
